# Integrative Analysis to Identify Genes Associated with Stemness and Immune Infiltration in Glioblastoma

**DOI:** 10.3390/cells10102765

**Published:** 2021-10-15

**Authors:** Neerada Meenakshi Warrier, Prasoon Agarwal, Praveen Kumar

**Affiliations:** 1 Department of Biotechnology, Manipal Institute of Technology, Manipal Academy of Higher Education, Manipal 576104, Karnataka, India; neerada.m.w@gmail.com; 2 KTH Royal Institute of Technology, School of Electrical Engineering and Computer Science, 10044 Stockholm, Sweden; 3 Science for Life Laboratory, 17121 Solna, Sweden

**Keywords:** integrative analysis, network analysis, DNA methylation, stemness, immune infiltration, GBM

## Abstract

It is imperative to identify the mechanisms that confer stemness to the cancer cells for more effective targeting. Moreover, there are not many studies on the link between stemness characteristics and the immune response in tumours. Therefore, in the current study involving GBM, we started with the study of BIRC5 (one of the rare genes differentially expressed in normal and cancer cells) and CXCR4 (gene involved in the survival and proliferation of CSCs). Together, these genes have not been systematically explored. We used a set of 27 promoter methylated regions in GBM. Our analysis showed that four genes corresponding to these regions, namely EOMES, BDNF, HLA-A, and PECAM1, were involved with BIRC5 and CXCR4. Interestingly, we found EOMES to be very significantly involved in stemness and immunology and it was positively correlated to CXCR4. Additionally, BDNF, which was significant in methylation, was negatively correlated to BIRC5.

## 1. Introduction

Glioblastoma multiforme (GBM) is the most malignant form of primary brain tumours in adults. This grade IV glioma is very aggressive and remodels the tumour microenvironment with the help of the immune system, stroma, and vasculature to survive. These tumours display high inter and intratumor heterogeneity and mutations in various genes and signalling pathways [1]. The current standard therapy for GBM includes maximal surgical resection, followed by concurrent radiotherapy and oral chemotherapy with temozolomide. This treatment regimen is followed by adjuvant chemotherapy by temozolomide and yet the median post-diagnosis survival period is around 15–18 months. Resistance to therapy and dismal prognosis are attributed to the extensive invasiveness, evasion of cell death, the protection of tumours by the blood–brain barrier, the heterogeneity of GBM, the lack of dependable targets, and most importantly, the immunosuppressive nature of the tumour and the presence of GBM cancer stem cells (GSCs) [2,3,4].

GSCs are subpopulation of cells within the tumour that self-renew and differentiate recapitulating the entire population of heterogenous cells in the tumour, resulting in tumour recurrence. Targeting GSCs are, hence, identified as one of the most significant therapeutic mechanisms against GBM. Direct targeting often includes targeting a range of surface or internal markers such as CD133, A2B5, CD44, CD15/SSEA, Sox-2, Nestin, OLIG2, NANOG, ALDH, Integrin-α6, KLF4, L1CAM. CXCR4, HIF-1α, OCT4, Survivin, GFAP, and SALL4. The second option is to target pathways assisting in the maintenance of stem cells and that include PI3K/Akt, Notch, Sonic Hedgehog, Stat3, NF-κB, Twist1-SOX2, N-Cadherin, and Wnt/β-Catenin signalling [5]. However, there is no single marker or pathway exclusive for targeting GBM CSCs. Hence, the best option would be to target two or more co-expressed markers that have the potential to target multiple CSC hallmarks [6,7].

Epigenetic reprograming is as important as genetic alterations in the development and progression of GBMs. DNA methylation, and both hyper and hypo methylation, are important biomarkers in these tumours and all the biologically relevant methylation occurs in the CpG islands in gene promoter regions. Hypermethylation is associated mainly with the activation of tumour suppressor genes, apoptosis, migration, invasion, DNA repair, and regulation of cell cycle. Hypomethylation activates oncogenes along with some tumour associated genes such as SOX-2, Oct-4, IGF-2, etc. [8,9]. Epigenetic orchestra is significant in the regulation of inter and intra tumoral heterogeneity in cancer that is usually associated with the CSC phenotype. It is also important in the reprogramming of transcription factors that promote stemness in GBMs [10]. Hence, deep understanding of the various hypo and hypermethylated genes in GBM and their association with various biomarkers involved in tumour progression would be effective in developing new treatment strategies.

CXCR4, is a G protein coupled chemokine receptor, involved in invasion and metastasis, epithelial mesenchymal transition (EMT), chemotaxis, angiogenesis, and CSC survival and proliferation. It is also associated with the regulation of immune cell infiltration in the CSC microenvironment, Hematopoietic and Mesenchymal Stem Cell recruitment, and homing in tumours [11]. The two ligands for the receptor are SDF-1/CXCL12 and Macrophage Migration Inhibitory Factor (MIF). The expression is usually high in tumour tissues and low in the normal tissue. Studies have revealed that its increased expression is often associated with mechanisms of EMT/CSCs induction [12]. Elevated expression often contributes to the differentiation of neural progenitor cells to neuronal precursors and helps is neurogenesis. The binding of CXCR4 to its ligand helps in the survival of Hematopoietic stem cells while negatively influencing their proliferation [13,14]. CXCR4 also stimulates many pathways such as PI3K/Akt, JAK/Stat, Wnt, TGF-β, SMAD, NF-κB, among others, in a variety of cancers, all of which play significant roles in CSC maintenance [15]. CXCR4 could be a good therapeutic target for directly targeting CSCs and effectively blocking recurrence. Studies have reported the overexpression of CXCR4 in GBM, where it mediates invasiveness, vascularization, and cell growth and maintenance and hence could be an indicator of poor prognosis [16].

Survivin (BIRC5), is a multifunctional oncofoetal gene with very little expression in normal adult differentiated tissues. It belongs to the class of inhibitor of apoptosis proteins (IAP) and plays important roles in the regulation of cell division, cell death, tumour growth and maintenance, and in CSC survival. Survivin is a downstream member of a set of signalling pathways including PI3K/Akt, Wnt/β-Catenin, TGF-β, JAK/STAT, NOTCH, among others. Most cancers show and increased expression of surviving, which is often associated with poor prognosis [17,18]. Higher expression of survivin has been reported in GBM, but it seems to increase in GSCs and has an effect on the immune reactivity and stemness along with metastasis, angiogenesis, and apoptosis in these tissues [19]. It plays a very prominent role in therapy resistance as well and, hence, is a very good direct target for targeting stem-like cells in GSCs [20].

## 2. Materials and Methods

Publicly available datasets were used for this integrative analysis. Study by Peng S et al., 2017 and Court F et al., 2019 (GSE123682) for DNA methylation in GBM versus normal and TCGA samples from UALCAN were used. For the gene expression, we used GBM datasets available in Oncomine, TCGA and CGGA. For single-cell analysis, we have used the study by Darmanis S et al., 2017. In Oncomine, we used the following search: Tumor type- GBM, Analysis type- Normal vs. Tumor and then used the gene name for the expression profile of that particular gene. The list of the final 27 differentially methylated regions (DMRs) is provided in the manuscript which was further used in various analyses.

### 2.1. Expression Profiles of BIRC5 and CXCR4

To understand the expression of BIRC5 and CXCR4 and to deduce their importance in cancer versus normal samples, we obtained the TCGA expression profiles of these genes with Timer 2.0 (http://timer.cistrome.org/, accessed on 14 October 2021) [21,22]. The Gene_DE module in the cancer exploration component was utilised for this. The differential expression is generated as box plot wherein the significance is determined using edgeR. Expression analysis in Oncomine was performed where cancer type was GBM, analysis type was cancer vs. normal analysis, and the genes that we checked for differential expression. The threshold p-value was 1E-4 and fold change greater the 2 and gene rank top 10% (https://www.oncomine.org, accessed on 14 October 2021) [23]. Chinese Glioma Genome Atlas (CGGA) database provides the differential gene expression profiles of genes between lower grades (WHO grade II and II) and higher-grade IV gliomas or GBMs (http://www.cgga.org.cn/about.jsp, accessed on 14 October 2021) [24]. We investigated the expression patterns of BIRC5 and CXCR4 in three different studies, mRNAseq_693, mRNAseq_325, and mRNA-array_301, within the database.

### 2.2. Role of Methylation in the Regulation of BIRC5 and CXCR4

We obtained the promoter methylation data of both the genes in TCGA GBM and normal samples using the UALCAN database (http://ualcan.path.uab.edu/, accessed on 14 October 2021) [25]. Promoter methylation levels of both these genes were obtained from the database as box plot. The methylation level is indicated by beta values ranging from 0 to 1 for unmethylated to fully methylated. The beta value between 0.7–0.5 is considered hyper methylated while those between 0.3–0.25 are hypomethylated. The methylation patterns of these genes were also searched in the CCGA database in the methyl_159 dataset. This was generated using Illumina Infinium HumanMethylation27 Bead-Chips.

We obtained the methylation data from Peng S et al., 2017 where the authors compared the long-term survival GBM patients with the short-term survival GBM patients [26]. We obtained a list of 67 regions that were differentially methylated between the LTS and the STS GBM patients. The annotation of the DMRs was done using Illumina HumanMethylation450_15017482_v1-2 array.

The differential methylated regions were obtained by GEOR using default parameters. The fdr was ≤0.05. Beta values used were estimated and represented by Court F et al., 2019 [27].

### 2.3. Functional Enrichment Analysis

Metascape was used for functional enrichment analysis and protein–protein interaction network analysis (http://metascape.org, accessed on 14 October 2021) [28]. We used custom analysis, keeping the default setting for enrichment analysis and used combined all as database setting for protein–protein interaction analysis. Molecular Complex Detection (MCODE) algorithm was used to select the densely connected network neighbourhood and this was performed in Cytoscape [29]. This neighbourhood component is more likely to be associated with a particular complex or functional unit than the rest of the network.

To understand the roles of the interacting proteins in tumours, stemness, and immunity, we performed string analysis of the genes in the network using 40 identifiers in Cytoscape. Functional enrichment analysis of the network components was performed in Metascape and Enrichr (https://maayanlab.cloud/Enrichr/, accessed on 14 October 2021) [30,31,32]. Enrichr is an interactive gene set enrichment web-tool with around 35 gene set libraries. In this analysis, GO biological process 2021, GO molecular function 2021, and GO cellular component 2021 data bases for gene ontologies and KEGG 2021 human, Reactome 2016, and Bioplanet 2019 gene libraries were used for pathway enrichment.

### 2.4. Validation of Expression and Methylation of EOMES, BDNF, PECAM1, and HLA-A

To validate our analysis, we obtained the expression profiles of these genes from TCGA using timer and CGGA databases, as described before. We also studied the methylation profile of these genes in CGGA database.

### 2.5. Role of Genes in Stemness

To understand the involvement of genes in stemness, we used StemChecker (http://stemchecker.sysbiolab.eu/, accessed on 14 October 2021) [33]. Here, the statistical significance (*p*-value) is calculated by hypergeometric test and is the significance of enrichment of genes, included in composite gene sets for the different stem cell types, stemness signature, and transcription factor among the input genes found. Adjusted *p*-value was calculated by Bonferroni correction.

### 2.6. Single Cell Expression of the Genes

We used http://www.gbmseq.org/, accessed on 14 October 2021) for understanding the expression pattern of genes in scRNAseq data in neoplastic cells, myeloid cells, vascular cells, neurons, oligodendrocytes, astrocytes, and OCPs. It consisted of scRNAseq data from a cohort of four primary GBM patients with IDH1 negative grade IV tumours [34].

### 2.7. Immune Infiltration Analysis

To perform immune infiltration analysis of the genes in TCGA-GBM, we used the Timer webtool. Using the gene module in immune association section, we obtained the immune infiltration levels of the genes across GBM using 8 different algorithms (CIBERSORT, CIBERSOR-ABS, XCELL, TIMER, QUANTISEQ, EPIC, MCPCounter, and TIDE). The purity adjusted spearman’s Rho for various cell types were obtained as immune cell types are negatively correlated to purity.

### 2.8. Correlation between Genes

We used correlation analysis platform from CGGA and Timer webtools to understand the correlation between BIRC5, CXCR4, and the differentially methylated genes in GBM.

### 2.9. Statistical Analysis

We have used default parameters in this study while using any tool.

## 3. Results

### 3.1. Gene Expression Profile of BIRC5 and CXCR4

**Figure 1:**
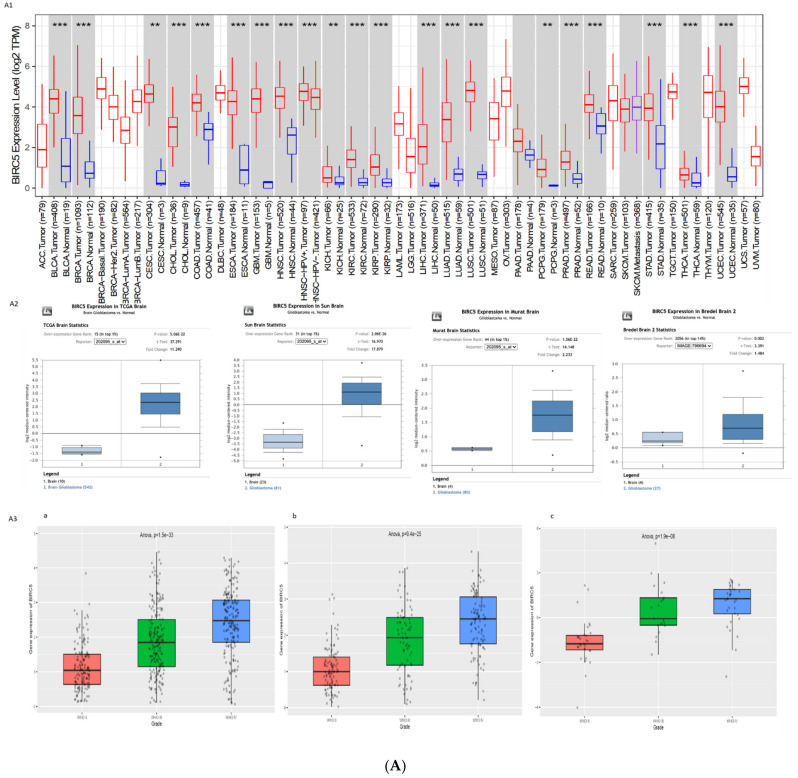

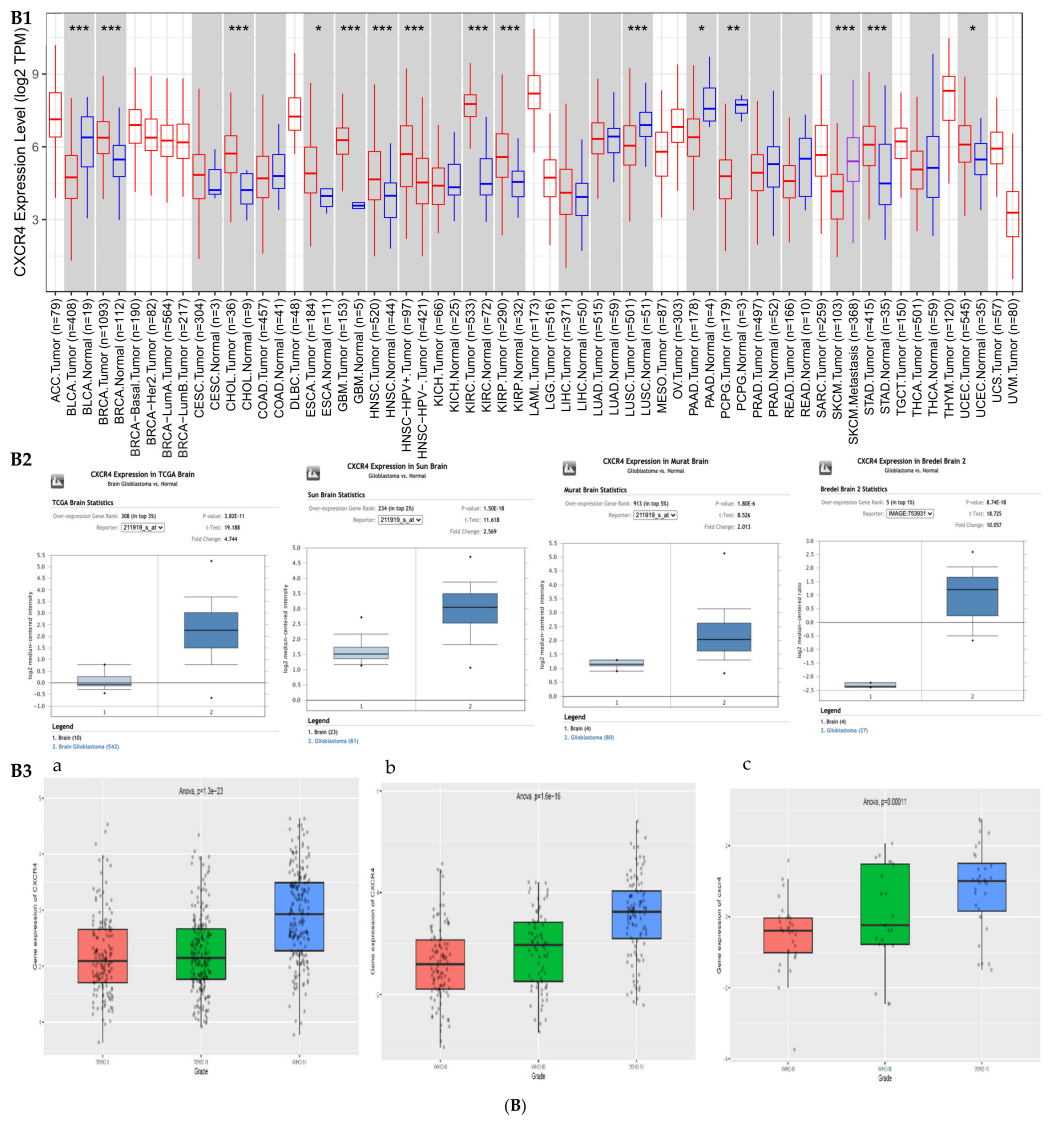
(**A**): Differential expression profiles of BIRC5. (**A1**) Across all cancer vs. corresponding normal tissue samples in TCGA data base using timer. (**A2**) Oncomine database across 4 different studies of GBM (**A3**) between WHO grade II, III, and IV Glioma samples from CGGA database (a) mRNAseq *n* = 693, (b) mRNAseq *n* = 325, and (c) mRNA-array *n* = 301. (**B**): Differential expression profiles of CXCR4. (**B1**) Across all tumour vs. corresponding normal tissue samples in TCGA data base using timer. (**B2**) Oncomine database across 4 different studies of GBM (**B3**) between WHO grade II, III, and IV Glioma samples from CGGA database (a) mRNAseq *n* = 693, (b) mRNAseq *n* = 325, and (c) mRNA-array *n* = 301. (*: *p*-value < 0.05; **: *p*-value < 0.01; ***: *p*-value < 0.001).

To understand the role of BIRC5 and CXCR4 in the GBMs, we assessed the expression profiles of the two genes in hundreds of GBM patients versus normal in different databases (TCGA, Oncomine, and CGGA). We found that the genes were differentially regulated in the several studies shown in Figure 1A,B. In the TCGA database, we looked for the differential expression of these genes in all the tumours and their corresponding normal tissues (Figure 1(A1,B1)). Both these genes were found to be over expressed in tumours in comparison to normal. Oncomine analysis showed significant upregulation of their expression in four different studies comparing normal and GBM samples (*n* = 542, 81, 80, and 27 samples) (Figure 1(A2,B2)). CGGA showed significant variation in expression between WHO grade II, III, and IV gliomas in two different RNA sequencing studies, mRNAseq (*n* = 693 samples) and mRNAseq (*n* = 325 samples), and one mRNAarray (*n* = 301 samples) (Figure 1(A3,B3)).

### 3.2. Epigenetic Regulation of BIRC5 and CXCR4

**Figure 2:**
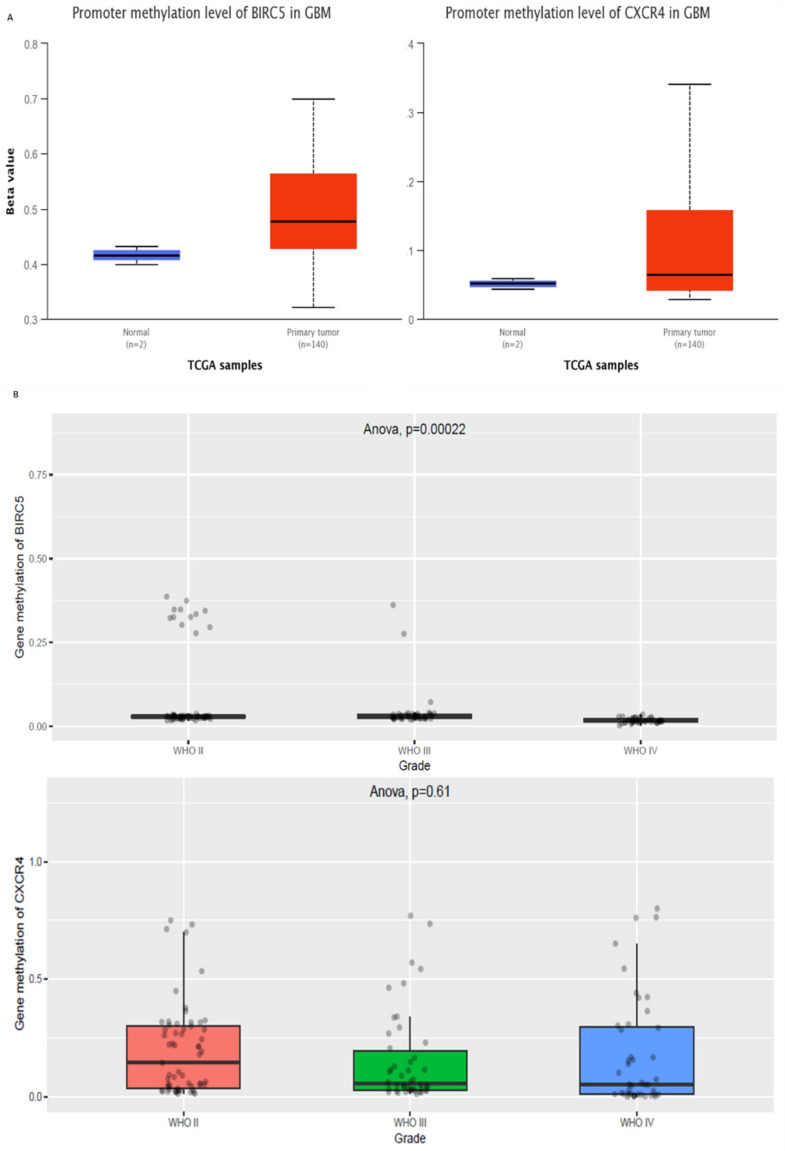
Methylation profile of BIRC5 and CXCR4. (**A**) Promoter methylation levels from TCGA normal and tumour samples (**B**) Methylation profile in different grades of glioma from CCGA database.

Subsequently, we wanted to study the role of epigenetics in the differential regulation of these genes. We obtained the promoter methylation pattern of these genes in TCGA samples from UALCAN (Figure 2A). Methylation profiles of these genes in *n* = 159 samples of WHO grade II, III, and IV gliomas from CCGA database were used in this analysis (Figure 2B). To understand the impact of epigenetically regulated genes in GBM, on the functions of BIRC5 and CXCR4, we used the study by Peng S et al., 2017 [26]. We obtained 67 differential methylated regions that were described in the Long-term survival (LTS) GBM vs. the Short-term survival (STS) GBM. These are the DMRs that are deregulated in high grade GBMs. To make things more comparable to gene expression, we selected 27 regions (Table 1) that were present up to 1500 bp upstream of the transcription start site (TSS), 5’UTR, 1st Exon, 3’UTR to their closest genes.

### 3.3. Epigenetic Regulation of Genes in Normal vs. GBM

Further to understand how the methylation vary in normal vs. GBM for the genes associated to DMRs obtained from the above study, we used another study by Court F et al., 2019 [27]. Using GEOR, we analysed the GBM samples (*n* = 27) versus the normal samples (*n* = 8) and found several DMRs that were significantly (adj value < 0.05) differentially methylated (*n* = 141971 DMRs) in GBM as compared to normal. Interestingly, in this analysis, there were several DMRs that were present in the study by Peng S et al., 2017. However, there were only two similar DMRs that were located in the promoter regions of the two genes EOMES and BDNF.

### 3.4. Functional and Network Analysis of Methylated Genes

**Figure 3:**
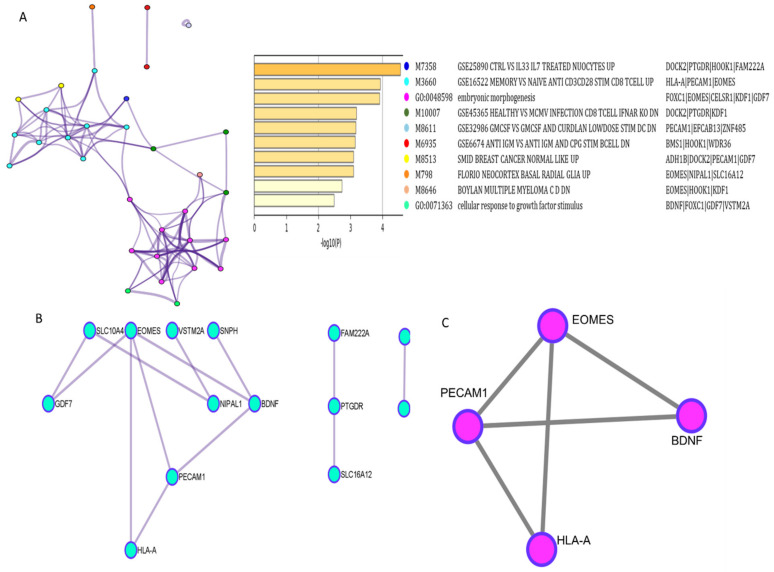
Functional enrichment and protein–protein interaction of 27 genes. (**A**) Functional Enrichment analysis of the 27 genes in promotor region obtained using Metascape represented as cluster with the heat map of the cluster based on P-value. (**B**) PPI network from Metascape analysis reproduced and edited in Cytoscape. (**C**) PPI network with highest MCODE scored from MCODE analysis in Cytoscape.

To understand the functional importance of these 27 DMRs, we performed pathway and process enrichment analysis on the nearest corresponding genes. Terms with a *p* value < 0.01, minimum enrichment of 1.5 and a minimum count of 3 overlapping genes are enriched to clusters. The analysis showed various immunological signatures such as control vs. IL33 IL7 treated Nuocytes UP, MEMORY VS. NAIVE ANTI CD3CD28 STIM CD8 TCELL UP, HEALTHY VS. MCMV INFECTION CD8 TCELL IFNAR KO DN, GMCSF VS. GMCSF AND CURDLAN LOWDOSE STIM DC DN, etc. GO biological processes embryonic morphogenesis and cellular response to growth factor stimulus and chemical and genetic perturbations SMID BREAST CANCER NORMAL LIKE UP, FLORIO NEOCORTEX BASAL RADIAL GLIA UP, BOYLAN MULTIPLE MYELOMA C D DN all of which are important in cancer progression in the top enriched clusters (Figure 3A). We found PECAM1, EOMES, DOCK2, CELSR1, HLA-A, KDF-1, PTGDR, BMS1, HOOK1, and GDF7 as the most frequent hits in many of the clusters.

To understand if these gene products have any interaction among themselves, we performed the protein–protein interaction analysis of these 27 promoter regions using Metascape. We used the MCODE algorithm in Cytoscape to identify densely connected network neighbourhood. We found a strong interaction between EOMES, PECAM1, BDNF, and HLA-A (Figure 3B). BDNF was identified as the seed by the MCODE algorithm and the MCODE score of the cluster was 3.333 (Figure 3C). Further, these genes were utilised to analyse their role in GBM and interaction with CXCR4 and BIRC5.

### 3.5. Validation of the Expression and Methylation Profiles of EOMES, BDNF, PECAM1, and HLA-A

**Figure 4:**
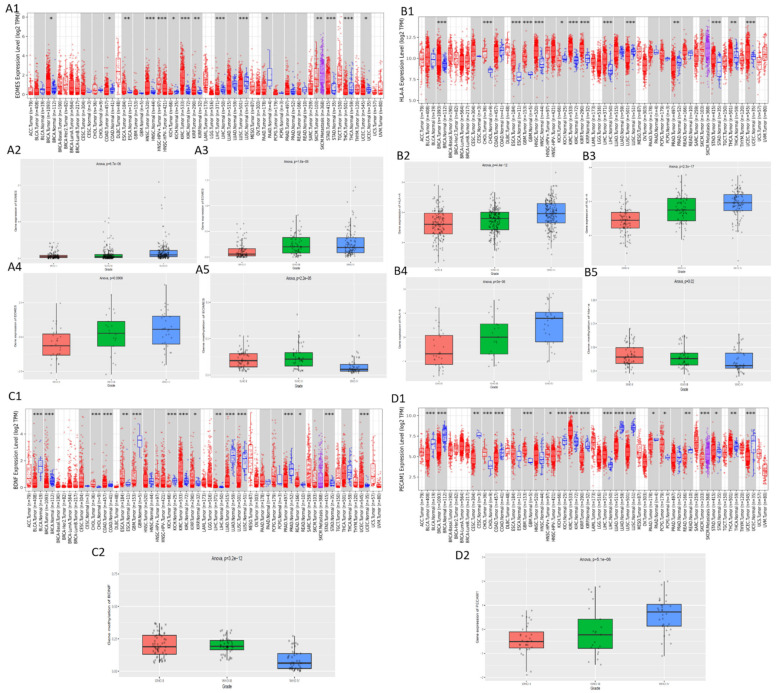
TCGA and CGGA expression profiles of EOMES, HLA-A, BDNF, and PECAM1. TCGA expression profiles of (**A1**) EOMES, (**B1**) HLA-A, (**C1**) BDNF, and (**D1**) PECAM1 in normal vs. tumor samples. CGGA expression profiles from mRNA seq (*n* = 693) (**A2**) EOMES and (**B2**) HLA-A, mRNA seq (*n* = 325) (**A3**) EOMES and (**B3**) HLA-A, mRNA-array (*n* = 301) (**A4**) EOEMS, (**B4**) HLA-A and (**D2**) PECAM1, and methylation (*n* = 159) (**A5**) EOMES, (**B5**) HLA-A and (**C2**) BDNF ((*: *p*-value < 0.05; **: *p*-value < 0.01; ***: *p*-value < 0.001).

To validate the expression profiles of these genes, we used Timer for TCGA (normal vs. tumour) and CGGA (between grade II, III and IV) tools (Figure 4). We observed a significant difference in the expression of HLA-A (Figure 4(B1)), BDNF (Figure 4(C1)), and PECAM1 (Figure 4(D1)) between normal and tumour samples. BDNF was found to be under expressed in tumour samples while the other two were higher in tumour samples. However, according to TCGA data, EOMES (Figure 4(A1)) showed no significant expression difference in GBM normal and primary tumour samples even though it showed upregulation in tumour samples. Interestingly, the expression profiles of EOMES displayed significant increase with increasing grades of tumours according to all the studies in CGGA database (Figure 4(A2–A4)). While BDNF was not significant in these samples, HLA-A showed significant upregulation (Figure 4(B2–B4)). PECAM1 expression as well was significant in one of the studies (Figure 4(D2)). Interestingly, the methylation data in CGGA showed significant difference in methylation of EOMES (Figure 4(A5)) and BDNF (Figure 4(C2)) between various grades of tumours. Even HLA-A showed significant variations in methylation (Figure 4(B5)). These profiles further add to the importance of these genes in GBM.

### 3.6. The Role of the Genes in Stemness

**Figure 5:**
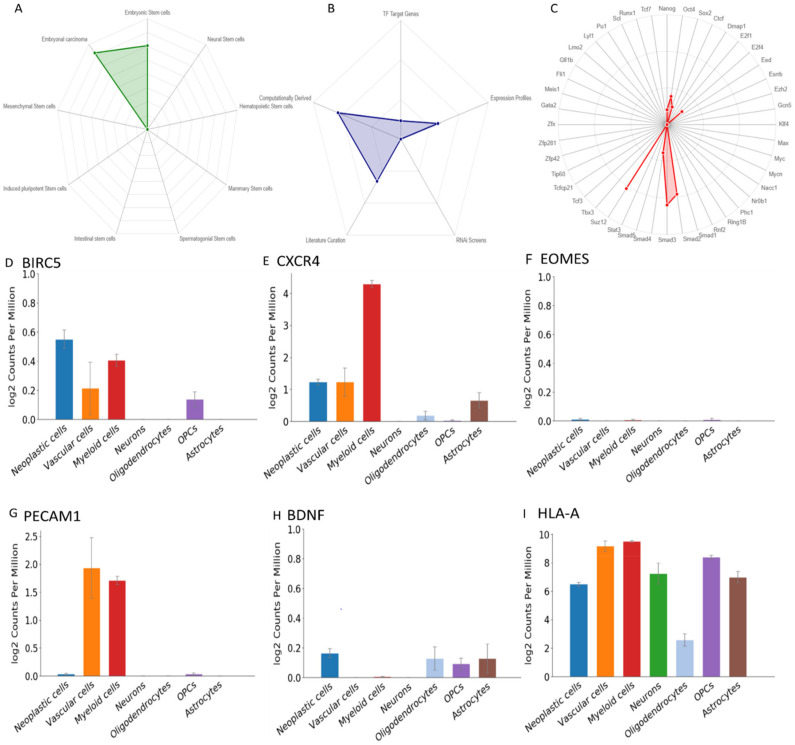
Expression of the genes in stemness and their expression profiles in single cell. Radar chart showing the overlap between (**A**) input genes and composite gene sets in stem checker with regard to stem cell types, (**B**) input genes, and stemness signatures. (**C**) Input genes and transcription factors. Overlap significance is plotted (–log 10 *p* value). Expression profiles of the hub genes in single cells obtained from GBM-seq (**D**) BIRC5, (**E**) CXCR4 (**F**) EOMES, (**G**) PECAM1, (**H**) BDNF, and (**I**) HLA-A.

Cancer is driven by cells having stem-like properties. Therefore, to understand the significance of these four genes together with BIRC5 and CXCR4 in stemness and tumour characteristics, we used the web tool StemChecker. The genes were compared to the software curated stemness signatures and various transcription factors linked to pluripotency and maintenance of stemness. These signatures consist of gene sets and transcription factors, associated with characteristics of stem cells. In this analysis, BIRC5 and EOMES were found to overlap in embryonic stem cells and embryonal carcinoma (Figure 5A). We observed BIRC5, EOMES, CXCR4, AND PECAM1 overlap with various stemness signatures including SOX2, NANONG, and OCT4 (Figure 5B). We also found that EOMES, BIRC5, and CXCR4 overlapped with transcription factors responsible for maintaining stemness and pluripotency. Out of this, the SMAD3_Kim was most significant with an FDR of <0.05 (Figure 5C).

### 3.7. ScRNA Expression Profile

To understand the specificity of the gene expression, i.e., in which cell type the gene is expressed, we analysed the expression of these genes in scRNA seq data of cohorts of four primary GBM patients of grade IV, IDH1-ve. The data constituted 3589 cells sequenced over 23,368 genes, from both tumour core and peritumoral brain tissues [34]. BIRC5 was predominantly expressed in neoplastic cells though some expression was observed in myeloid cells (Figure 5D). The expression of CXCR4 was found to be highest in myeloid cells, with lesser expression in neoplastic cells and vascular cells (Figure 5E). EOMES expression was absent or very low in almost all cell types (Figure 5F), while PECAM1 was expressed by vascular cells and myeloid cells (Figure 5G). Low expression of BDNF was found in neoplastic cells, oligodendrocytes, OCPs, and astrocytes (Figure 5H) and HLA-A showed high expression in almost all cell types excluding oligodendrocytes (Figure 5I). Thus, this suggests that these genes have varied expression profiles in different cell types.

### 3.8. Immune Infiltration

**Figure 6:**
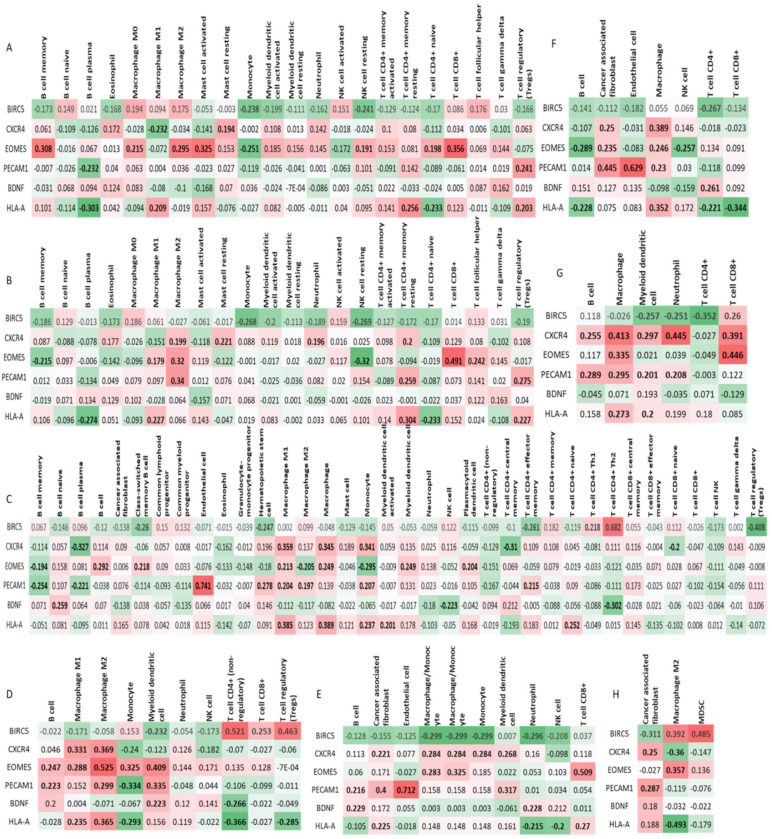
Timer analysis heatmaps of 7 algorithms for the 6 genes (**A**) CIBERSORT, (**B**) CIBERSORT-ABS (**C**) XCELL (**D**) QUANTISEQ (**E**) MCPCounter (**F**) EPIC (**G**) TIMER, and (**H**) TIDE. *p* < 0.05 is given in bold.

Since immune cell infiltration is significant in tumour development and progression, we wanted to estimate the immune infiltration of the six genes using multiple immune deconvolution methods (Figure 6A–H). We correlated the genes to T Cell CD8+, T cell CD4+, Regulatory T cell, T cell follicular helper, T cell gamma delta, T cell regulatory, B cells, Neutrophils, Monocytes, Macrophages, Dendritic cells, natural killer cells, mast cells, Endothelial cell, Eosinophil, Cancer associated fibroblasts, and hematopoietic stem cells in GBM. Interestingly, all the genes were positively or negatively correlated to different cell types. PECAM1 was significantly positively correlated to endothelial cells. EOMES was significantly correlated to several cell types, while BDNF was the least correlated gene. PECAM1, CXCR4, and EOMES were mostly positively correlated to several cell types. BIRC5 was mostly negatively correlated to several cell types. Thus, this suggests that these genes have a good immune infiltration in GBMs.

### 3.9. Protein–Protein Interaction

**Figure 7:**
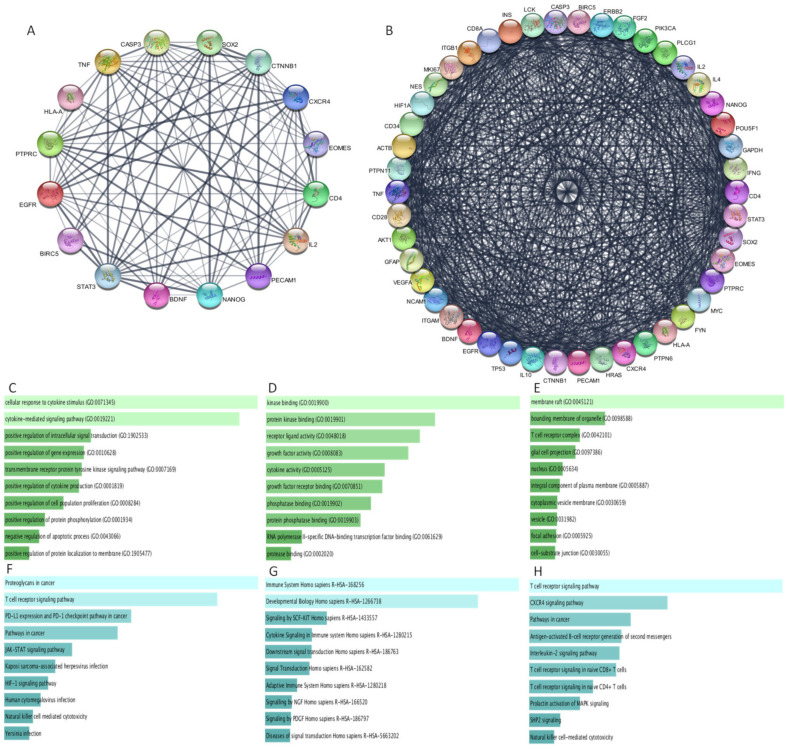
Protein–protein interaction and functional enrichment analysis of BIRC5, CXCR4, EOMES, BDNF, HLA-A, and PECAM1. (**A**) String analysis of CXCR4, BIRC5, BDNF, PECAM1, EOMES, and HLA-A with 10 and (**B**) with 40 identifiers performed in Cytoscape. (**C**–**H**) Bar Graph from Enrichr based on *p*-value ranking (**C**) GO Biological Processes (**D**) GO Molecular function (**E**) GO cellular component (**F**) KEGG human 2021 (**G**) Reactome 2016, and (**H**) Bioplanet 2019.

We next wanted to understand how the six genes interact with each other and their role in tumour maintenance. The genes were further subjected to String database analysis in Cytoscape with both 10 and 40 identifiers to ascertain the other proteins interacting with these genes (Figure 7A,B). With 10 identifiers, we observed that these genes interacted with stemness genes such as SOX-2 and NANOG, genes important in cancer pathways such as STAT3, CTNNB1, EGFR, TNF, and CASP3, and immune genes such as IL2 and CD4. This provides the importance of these genes in these processes. Additionally, to obtain a better perspective of more interacting partners and the potential pathways and processes, we performed string analysis with 40 identifiers. Here, we identified that these genes are also linked to stemness genes such as NESTIN, POUF51, CD34, and GFAP and immune related genes including IL4, ITGB1, ITGAM, IL-10, IFN-γ, and CD28. Genes including TP53, AKT1, MYC, HIF1A, and PI3KCA that play significant role in various cancer processes were also interacting with these 6 genes, thus indicating that these 6 genes significantly interact with several key genes that are involved in the tumour characteristics.

### 3.10. Functional Analysis of Protein–Protein Interaction Network

To understand how the interacting genes were functionally important, we performed enrichment analysis of the 46 genes in Enrichr. The top enriched biological processes included cytokine mediated signalling pathway, cellular response to cytokine stimulus, positive regulation of cell proliferation, positive regulation of gene expression, negative regulation of apoptosis, etc. (Figure 7C). BIRC5 and CXCR4 along with ITGB1, ITGM, IL-10, Stat 3, IL-4, IL-2, IFNG, Nanog, SOX2, VEGFA, HIF1A, TP53, MYC, etc., were the most frequent genes in the top enriched biological processes. PECAM1 and BDNF were also involved in some of the top processes. The GO molecular function 2021 showed kinase binding, protein kinase binding, growth factor activity, receptor ligand activity, growth factor receptor binding, and cytokine activity, among others, as highly enriched, while BDNF was involved in many of the processes along with immune related genes such as IL10, IL2, IL4, and IFNG (Figure 7D). PECAM1, HLA-A, and BIRC5 along with ITGB1, CD8A, EGFR, CD4, and PTPRC were some of the overlapping genes in the GO cellular component database (Figure 7E).

Pathway analysis with KEGG human (Figure 7F), Reactome (Figure 7G), and Bioplanet (Figure 7H) databases showed many significant cancer, stemness, and immune signalling pathways. Proteoglycans in cancer, pathways in cancer, T-cell receptor signalling pathways, PD-L1 expression and PD1 checkpoint pathways, Jak-Stat signalling pathway, HIF-1 signalling pathway, and PI3K-Akt signalling pathway were among the top enriched pathways in KEGG. Reactome database enriched pathways aiding in developmental biology homo sapiens, immune system homo sapiens, cytokine signalling inmune system homo sapiens, etc. The pathways enriched in Bioplanet were the same as the ones enriched in KEGG and Reactome. Thus, this analysis indicates that all the interacting genes are involved in the various important biological processes and thus are key for the tumour biology. Enrichment tables are given in Supplementary Table S1.

### 3.11. Correlation between BIRC5, CXCR4, and Methylated Genes

**Figure 8:**
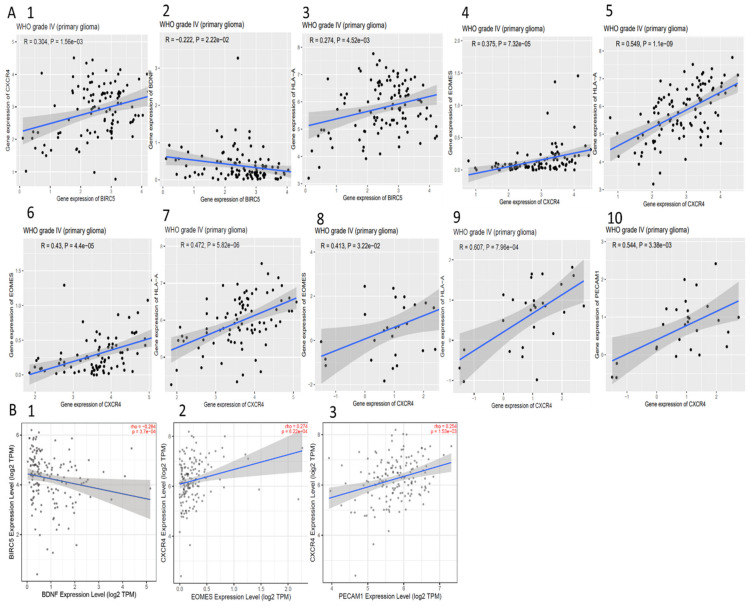
Coexpressions of BIRC5 and CCR4 with each other and with the methylated genes from CGGA and TIMER (for TCGA). (**A**) (**1**–**5**) mRNA_693, (**6**,**7**) mRNA_325, (**8**–**10**), and mRNA_Array_301 show the significant (*p* < 0.05) correlation between the genes from timer, (**B**) (**1**–**3**) shows the significant (*p* < 0.05) correlation from Timer.

We used the correlation analysis platform from CGGA database (Figure 8A) and TIMER (Figure 8B) to understand if BIRC5 and CXCR4 expressions are correlated with each other and/or with the other four genes in the GBMs. We observed that BIRC5 was significantly negatively correlated with BDNF in primary GBM in the CGGA database and the TCGA database (Figure 8(A2,B2)). The CGGA database also showed positive correlation between BIRC5 and CXCR4 as well as BIRC5 and HLA-A in primary GBM (Figure 8(A1,B3)). Interestingly, almost all the studies showed positive correlation of CXCR4 with EOMES as well as HLA-A (Figure 8(A3–A9,B2)). A significant relationship was also observed with PECAM1 in one of the studies in CGGA and TCGA datasets (Figure 8(A10,B3)). Thus, this analysis clearly indicates that expression profiles of these genes is significantly correlated.

## 4. Discussion

In this study, we observed the expression, interaction, and roles of CXCR4 and BIRC5 in GBM tumour samples in relation to epigenetics, stemness, and immunity. These genes were highly expressed in GBMs as compared to normal brain in different databases, indicating their importance in cancer and potential as therapeutic targets. We did not observe any significant difference in the promoter methylation patterns of BIRC5 and CXCR4 between normal and GBM samples in different databases. It has been studied that the correlation between BIRC5 promoter methylation and expression is complicated in cancers. However, methylation profiles have a significant influence in the regulation of the expression of BIRC5 in many cancers [35,36]. On the other hand, it has been observed that CXCR4 expression in GBM is regulated by DNA methylation and hypermethylation is often associated with better survival [37].

To understand the influence of promoter methylation of interacting genes on these two genes, we used the DNA methylation data for long term and short-term survival in patients with GBM by Peng et al., 2017. Epigenetic modifications are important in the regulation of tumour heterogeneity, CSC survival, and maintenance in GBM. Altered gene expression between stem cells and tumour cells have been attributed to differentially expressed and methylated promoter genes. DNA methylation status in specific promoters is responsible for immune surveillance [38]. There is a significant difference in epigenetic profiles within various grades of glioma as well. Promoter hypermethylation is usually associated with tumour suppresser gene inhibition while hypomethylation often activates oncogenes [39,40]. Hence, understanding epigenetic modifications in tumour cells can help in the early diagnosis and development of therapeutic strategies by influencing genes and transcription factors helping in stem cell maintenance and immune suppression. Of the 67 differentially methylated regions observed, we used the ones located in the 5′UTR, 3′UTR, first exon and 1500 bp upstream of TSS, region in our study. On comparing the epigenetic alterations of these genes in the normal and tumour sample data from Court F et al., 2019, we observed many of the DMRs overlapping with that of Peng et al., 2017. The same probes of EOMES and BDNF found in both the datasets could be indicative of the involvement of these methylations in these regions in GBM. It is known that DNA methylation of EOMES is an important biomarker in medulloblastoma, bladder, and liver cancers [41]. Similarly, the role of BDNF in various cancer processes have been reported as well. Its interaction with TRKB is important in various cancer and CSC pathways [42,43]. However, to the best of our knowledge, in the case of GBM, the role of these two genes is unexplored.

EOMES, PECAM1, HLA-A, and BDNF formed a MCODE network on performing PPI analysis. These gene were predominantly involved in immune system and developmental processes. We further used these four genes along with CXCR4 and BIRC5 in the all-downstream analysis. Our stemness analysis validated the role of BIRC5 in the development and maintenance of embryonic stem cells and embryonal carcinomas. It also suggested its interaction with SOX-2 and OCT 4. Studies have shown the involvement of Oct-4 and/or Sox-2 in regulating BIRC5 expression in embryonic stem cells and neural stem cells. Sox-2 mediated upregulation of BIRC5 is important in tumour progression, apoptotic inhibition, and resistance to chemotherapy in cancers [44]. Single cell analysis showed increased expression in neoplastic cells and myeloid cells suggesting the multifunctional role in cancer. EOMES was found to be positively correlated with most of the immune cell types in our timer analysis using various algorithms. The expression profile and methylation pattern of the gene was also significant in different grades of tumours in CGGA data, although the high expression observed in tumours was not significant in comparison to normal samples as per TCGA data. The literature also suggests that it is expressed highly in various subset of T cells and resting and activated NK cells. It is also important in inducing central memory T cells, repressing memory stem T cells and in differentiation of T cell subtypes [45,46]. At the tissue level, EOMES is responsible for inhibition of antitumour immune response resulting from T cell exhaustion and controlling the expression of IFN-γ, CXCR3, and CXCR4, while in the peripheral lymphoid system, they promote adaptive antitumour immune responses [47]. The level of EOMES expression might be indicative of progressive differentiation and maturation of IFN-γ-producing Th1-like γδ T cells wherein its expression is regulated by various interleukins [48]. This gene is also significant in the differentiation of human embryonic stem cells to mesoderm and endoderm [41]. Interestingly, our single cell analysis suggests a very low expression profile of EOMES in all the cell types that are involved in GBM. However, it will be interesting to perform single cell analysis of immune cells in GBM to have a better understanding of its role in the disease biology. CXCR4 was found to be involved with stemness signatures, which substantiates its role as stemness marker. It was highly expressed in vascular endothelial cells, neoplastic cells, and myeloid cells as per single cell analysis, attesting its role in angiogenesis and metastasis in cancer. This gene was related significantly to many immune cell types which validated its role in regulating B cell, T cell, Tregs, NK cells, Macrophages, DCs, Neutrophils, progenitor cells, vascular endothelial cells, myeloid suppressor cells, and microglia [49]. PECAM1 predominantly showed positive correlation with majority of immune cell types and was highly expressed in vascular and myeloid cells. Reports suggest that it is upregulated in platelets, endothelial cells, leukocytes, and T cells in various cancers. It is significant in the vascular development and migration of these cells and involved in cancer survival, invasion, and metastasis [50,51]. PECAM1 along with Nestin was found to be significantly expressed in GBM and GSCs and was important in chemoresistance and prognosis [52]. In our analysis, BDNF was found to be down regulated in tumours, while HLA-A was upregulated in tumours. BDNF and HLA-A showed significant difference in methylation between various grades of tumours, but were not involved in stemness, according to our analysis. They were positively correlated with many immune cell types, but their expressions in single cells were not conclusive of their role in cancer.

On the string network analysis of these genes with 40 identifiers, we observed that BDNF, HLA-A, EOMES, PECAM1, and CXCR4 interacted directly with each other with an MCODE score of 4.55 (Supplementary Figure S1). BIRC5 was found to interact with all the other five through SOX2. The network showed possible connections of these genes with a myriad of genes involved in various cancer signalling pathways, stem cell development, and maintenance and regulation of the immune system. STAT3, PIK3CA, Akt1, VEGFA, EGFR, TP53, MYC, HIF-1A CTNNB1, TNF, FYN, and Caspase 3 were genes significant in the regulation of cell cycle, cell proliferation, differentiation, migration, angiogenesis, and both apoptotic and autophagic cell death. Immune system regulators interacting with the genes included Interleukins-2, 4 and 10, IFNG, Lck, TNF, ITGB1, ITGAM CD4, and CD28. We also observed cell adhesion molecules (CAMs) responsible for cell migration, protein tyrosine kinases (Src, PTPN11 and PTPN6), and stem cell makers such as POU5F1 (OCT4), SOX2, Nestin, GFAP, Nanog, and CD34 in this network. It was observed that BIRC5 was mostly involved with cell proliferation and cell death related genes along with genes involved in stemness. On the other hand, EOMES was mostly involved with stemness signature and immune regulators. We observed that SOX2, NANOG, and STAT3 were interacting with all these genes, hence could be responsible for their regulation and the correlated expression patterns observed in various studies. EOMES overexpression in colorectal cancers was found to be important in the reciprocal regulation of BIRC5 and pro-apoptotic Bcl-2 modifying factor [53]. PECAM1 regulates cell proliferation and apoptosis with the help of CD44 and this interaction has a significant effect on the BIRC5 mediated regulation of caspases [54]. CXCR4 upregulates Survivin via PI3K pathways, which in turn mitigates radiation induced apoptosis in cancer cells. PECAM1 also enhances the CXCR4 mediated chemotaxis by the activation of PI3K/Akt/mTOR pathway [55]. Enrichment analysis of the 46 genes in the string network further supported these interactions and the respective pathways that regulates them. Cytokine mediated signalling pathways, apoptosis process regulation, regulation of cell proliferation, regulation of cell migration, and various other biological processes significant in cancer progression and recurrence were enriched in this analysis. These genes were also active in pathways in cancer, immune signalling, cytokine signalling, signalling pathways like JAK/Stat, HIF-1, IFN-γ, IL-2, and PD-L1, among various others. CXCR4, BIRC5, EOMES, and PECAM1 were very frequent hits in most of these pathways and processes. Correlation analysis based on the gene expression suggests a positive significant correlation between EOMES and CXCR4 and PECAM1, and CXCR4. These were in agreement with the reported coexpression patterns. A negative significant correlation between BIRC5 and BDNF expression profile suggests an involvement of epigenetically controlled BDNF in regulating BIRC5 in GBM. This provides an insight into the various roles played by these genes in tumour initiation and progression, generation of immune responses, and their potential as therapeutic targets. The interactions between these genes and the pathways that control them is also important in designing better therapeutic strategies that can target multiple cancer mechanisms simultaneously.

In our bioinformatics analysis, we have used various resources and tools to understand the importance and role of BIRC5 and CXCR4 in thousands of GBM samples. With our integrative analysis, we were able to identify the genes that were interacting with BIRC5 and CXCR4. We were able to identify genes like BDNF and EOMES that are epigenetically regulated and play significant role in stemness and immune related processes, to interact with the two genes. Our analysis suggests that EOMES strongly interacts with several well established stemness, immune, and cancer markers. However, there are limitations of our study, where we have used only the publicly available tools and datasets in this analysis. For future direction, we suggest that studies which include single cell immune analysis in GBMs will help in better understanding the role of EOMES, BIRC5, and CXCR4.

## Figures and Tables

**Table 1:** The list of 27 differentially methylated genes located up to 1500 bp upstream of transcription start site (TSS), 5′UTR, 1st Exon, 3′UTR to their closest genes.

Probe Name	Nearest Gene Symbol	*p* Value	Adjusted *p* Value	Beta Difference	UCSC_Ref_Gene_Group	Relation to UCSC_CpG_Island
cg00236919	NIPAL1	0.0266	0.0266	−1.6098	TSS1500	N_Shore
cg02742775	C12orf34	0.0062	0.0062	1.48821	5′UTR	Island
cg05454446	BMS1	0.0044	0.0044	−1.4252	TSS200	N_Shore
cg08085267	C17orf57	0.0031	0.0031	−1.9814	5′UTR	S_Shore
cg08209133	SLC10A4	0.0031	0.0031	−1.464	1stExon	Island
cg12297409	ZNF485	0.0003	0.0003	−1.5062	TSS200	N_Shore
cg18624900	SLC16A12	0.0205	0.0205	−1.6339	TSS1500	Island
cg19868631	VSTM2A	0.0343	0.0343	1.45488	TSS1500	Island
cg24368912	ADH1B	0.0031	0.0031	1.48875	TSS1500	
cg01912921	FOXC1	0.0085	0.0085	−1.6797	1stExon	Island
cg08699174	PECAM1	0.0205	0.0205	2.181	TSS1500	
cg11585022	WDR36	0.0205	0.0205	1.44761	TSS1500	N_Shore
cg11718030	BDNF	0.0117	0.0117	−1.3965	TSS1500	Island
cg11792516	HOOK1	0.0014	0.0014	−1.5621	TSS200	N_Shore
cg15308664	EOMES	0.0117	0.0117	−1.7284	TSS200	Island
cg17341969	SNPH	0.0266	0.0266	1.62061	3’UTR	S_Shore
cg18552861	GDF7	0.0205	0.0205	−1.4586	TSS1500	Island
cg19585676	HLA-A	0.0434	0.0434	1.53707	3’UTR	S_Shore
cg21070172	CELSR1	0.0343	0.0343	−1.4559	1stExon	Island
cg23022053	PTGDR	0.0266	0.0266	1.63417	TSS1500	N_Shore
cg26503653	DOCK2	0.0009	0.0009	−1.496	TSS1500	N_Shore
cg27049594	OR8A1	0.0205	0.0205	2.28489	TSS1500	
cg08231348	HOOK1	0.0266	0.0266	−1.586	TSS200	N_Shore
cg10706454	C1orf172	0.0205	0.0205	−1.5193	TSS200	Island
cg15533046	C12orf34	0.0117	0.0117	1.4762	5’UTR	N_Shore
cg25340688	MIR886	0.0266	0.0266	−1.5062	TSS200	Island
cg25502144	NCRNA00028	0.0044	0.0044	−1.5589	TSS200	S_Shore

## Data Availability

All the data used in this study were drawn from public databases and were permitted for use. TCGA, CGGA and Oncomine data were obtained from (http://timer.cistrome.org/, accessed on 14 October 2021, (http://ualcan.path.uab.edu/, accessed on 14 October 2021), (http://www.cgga.org.cn/about.jsp, accessed on 14 October 2021) and (https://www.oncomine.org, accessed on 14 October 2021). The first dataset used for methylation difference between LTS and STS was obtained from EMBL-EBI European Nucleotide Archive database (http://www.ebi.ac.uk/ena/, accessed on 14 October 2021) with accession number PRJEB10881 where Methylation data are available with accession number ERS1205964. The second data set for validation of methylation between normal and GBM was downloaded from NCBI Gene Expression Omnibus (GEO; https://www.ncbi.nlm.nih.gov/geo/, accessed on 14 October 2021) under the following accession number GSE123682.

## Supplementary Materials

The following are available online at https://www.mdpi.com/article/10.3390/cells10102765/s1, Figure S1: Interaction between CXCR4, EOEMS, BDNF, PECAM1 and HLA-A; Table S1: Enrichment analysis of PPI with 40 identifiers.

## Abbreviations

BIRC5	Baculoviral IAP Repeat Containing 5
CXCR4	C-X-C Motif Chemokine Receptor 4
EOMES	Eomesodermin
BDNF	Brain derived Neurotrophic Factor
HLA-A	Human Leukocyte Antigen A
DMRs	Differentially methylated regions
PECAM1	Platelet Endothelial Cell Adhesion Molecule 1
GBM	Glioblastoma Multiform
CSCs	Cancer Stem Cells
GSCs	Glioblastoma Stem Cells
TCGA	The Cancer Genome Atlas
CGGA	Chinese Glioma Genome Atlas
SDF-1	Stromal Cell-Derived Factor
Sox-2	SRY (Sex Determining Region Y)-Box 2
Oct-4	Octamer-Binding Transcription Factor 4
HIF	Hypoxia Inducible Factor
JAK	Janus Kinase
Stat	Signal Transducer and Activator of Transcription
VEGF	Vascular Endothelial Growth Factor
PI3K	Phosphoinositide-3 Kinase
EGFR	Epidermal Growth Factor Receptor
IGF	Insulin-like Growth Factor
mTOR	Mammalian Target of Rapamycin
DOCK2	Dedicator Of Cytokinesis 2
CELSR1	Cadherin EGF LAG Seven-Pass G-Type Receptor 1
KDF-1	Keratinocyte Differentiation Factor 1
PTGDR	Prostaglandin D₂ receptor
GDF7	Growth Differentiation Factor 7
NF-ĸB	Nuclear Factor Kap-pa-Light-Chain-Enhancer of Activated B Cells
TGF-β	transforming growth factor-β
L1CAM	L1 Cell Adhesion Molecule
SALL4	Sal-like protein 4
OLIG2	Oligodendrocyte Transcription Factor 2
GFAP	Glial fibrillary acidic protein
KLF4	Kruppel-like factor 4
IL	Interleukin
ALDH	Aldehyde dehydrogenase
IFN	Interferon
ITG	Integrin
PTPN	Protein Tyrosine Phosphatase Non-Receptor
CTNNB1	Catenin beta-1
PTPRC	Protein Tyrosine Phosphatase Receptor Type C
PD-L1	Programmed death-ligand 1
TNF	Tumour necrosis factor
DC	Dendritic Cells
NK cells	Natural Killer cells
TH1	Type 1 T helper
