# Supplementary material for: Integrative Analysis to Identify Genes Associated with Stemness and Immune Infiltration in Glioblastoma
Source: Cells. 2021 Oct 15;10(10):2765. doi: 10.3390/cells10102765 (PMC8534801; doi:10.3390/cells10102765)
Supplement: Supplementary file 1 [file cells-10-02765-s001.zip › cells-1418988-supplementary.pdf]

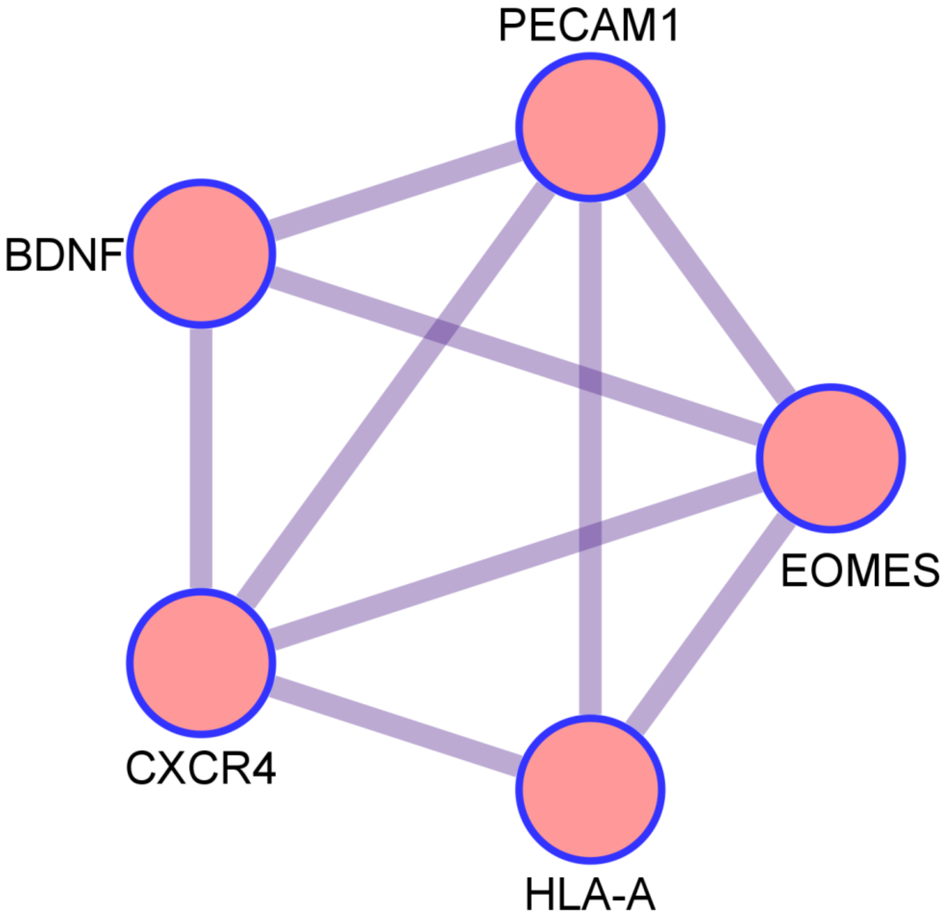

Supplementary Figure S1: Protein-Protein interaction between CXCR4, EOEMS, BDNF, PECAM1 and HLA-A with MCODE score 4.55 generated using Cytoscape.
